# Debonding Behavior of Resin-Cemented Attachment-Housing Denture-Base Complexes Under Cyclic Mechanical Loading

**DOI:** 10.3390/ma19112246

**Published:** 2026-05-26

**Authors:** Evangelos V. Skondras, Savvas N. Kamalakidis, Eftychia Skondra, Antonios Bouzakis, Lambrini Papadopoulou, Eleana Kontonasaki, Olga Naka

**Affiliations:** 1 Department of Prosthodontics, School of Dentistry, Faculty of Health Sciences, Aristotle University of Thessaloniki, 54124 Thessaloniki, Greece; eskondras13@hotmail.com (E.V.S.); drkamalakidis@dent.auth.gr (S.N.K.); kont@dent.auth.gr (E.K.); 2 Department of Prosthodontics, Tufts University School of Dental Medicine, Boston, MA 02111, USA; 3 Skondras Dental Center, 14671 Kifissia, Greece; skondraevi@yahoo.gr; 4 Impact-BZ, London SW11 5QL, UK; a.bouzakis@impact-bz.com; 5 Department of Geology, Schools of Sciences, Aristotle University of Thessaloniki, 54124 Thessaloniki, Greece; lambrini@geo.auth.gr

**Keywords:** attachment housing, cyclic loading, debonding, denture framework, PEEK, PMMA

## Abstract

Implant-supported overdentures improve denture retention and patient satisfaction, but debonding of attachment housings from the denture base remains a frequent prosthetic complication. This in vitro study evaluated the influence of attachment-housing and denture-base materials on debonding occurrence and maximum tensile force in resin-cemented attachment housing denture-base complexes subjected to cyclic mechanical loading. Thirty standardized specimens were digitally designed and fabricated from three denture-base materials—polymethylmethacrylate (PMMA) (*n* = 12), polyetheretherketone (PEEK) (n = 12), and cobalt-chromium (Co-Cr) (n = 6)—and combined with either titanium or PEEK attachment housings, which were bonded with a dual-polymerized resin cement. The specimens were subjected to 1100 cycles of alternating tensile and compressive loading, and debonding occurrence and maximum tensile force were recorded. Debonding occurred in 60% of the specimens and differed significantly among denture base materials. No debond-ing was observed in the Co-Cr specimens, whereas debonding occurred in 75% of the PMMA and PEEK specimens. The Co-Cr specimens also demonstrated significantly higher maximum tensile force values than the PMMA and PEEK groups, while regarding the attachment-housing material, no significant main effect was detected. Within the limitations of this in vitro study, the denture-base material, fabrication, and surface treatment combinations significantly influenced debonding and tensile force during cyclic loading, whereas the attachment-housing material did not demonstrate a significant main effect.

## 1. Introduction

Implant-supported overdentures (IODs), irrespective of their attachment mechanism, have been associated with higher patient oral-health-related quality-of-life and satisfaction scores than conventional removable complete dentures [1]. Several IOD attachment systems, including stud-type, bar, telescopic, and magnetic systems, have been developed to improve denture support and retention and to aid masticatory force distribution [2]. Retentive insert replacement has been reported as the most common prosthetic complication, followed by intraoral implant abutment replacement and prosthesis fracture [3]. Areas of reduced acrylic base thickness adjacent to or at the top of the attachment housings have been identified as fulcrum points that lead to fractures, requiring either prosthesis repair or replacement [4].

To overcome the inherent structural deficiencies of resin-based IODs, various reinforcement materials, primarily metallic alloys and polymeric fibers, have been introduced to improve mechanical properties and reduce fracture incidence. In addition, metallic reinforcement frameworks have been shown to decrease denture base deformation and reduce tensile stresses adjacent to the attachment housing by more than 60% when compared to non-reinforced bases [5]. Furthermore, it has been reported that denture-base deformation transmits compressive forces to the underlying residual ridge and the implant attachment system, thereby accelerating residual ridge resorption and increasing strain on the supporting elements of the overdenture prosthesis. These forces could be reduced and distributed more evenly by embedding rigid metallic reinforcement frameworks in the denture bases, thereby improving their long-term performance [6].

Recent advances in computer-aided manufacturing and computer-aided design (CAD-CAM), alongside the introduction of contemporary denture base and framework materials, have expanded the laboratory fabrication options for IODs. These newly developed materials have also necessitated enhanced adhesive protocols, including the use of dedicated primers and surface-modification treatments [7,8]. High-performance polymeric materials, primarily polyetheretherketone (PEEK), have been marketed for numerous dental applications, including the construction of overdenture frameworks [9,10]. IODs incorporate various attachment housing designs to facilitate retention [11]. These housings can be activated (pick-up procedure) intraorally using various securing (orientation) materials, with polymethylmethacrylate (PMMA) acrylic resins being the most popular [12]. To minimize the metallic appearance of titanium attachment housings and improve biocompatibility, manufacturers have introduced PEEK attachment housings [13]. The debonding effects between the attachment housing and the denture base or framework have been investigated in numerous in vitro studies [14,15,16,17,18,19,20], most of which have employed monotonic push-out or shear testing protocols to determine the maximum bond strength. However, limited information exists on the interfacial connection between the IOD components, particularly regarding resin-cemented attachment-housing denture-base complexes. Specifically, as the clinical failure of attachment housings is more likely to occur after repeated insertion–removal cycles and functional loading rather than under a single catastrophic load, fatigue-oriented evaluation may provide more clinically relevant information.

The aim of this in vitro study was to evaluate the effects of attachment-housing and denture-base materials on the occurrence of debonding and on the maximum tensile force measured during cyclic mechanical loading for different resin-cemented attachment-housing denture-base complexes. The null hypothesis was that no significant differences would be detected among the attachment-housing materials, denture-base materials, or their combinations with respect to debonding occurrence and maximum tensile force during cyclic mechanical loading.

## 2. Materials and Methods

Three denture base materials (two polymeric and one metallic) and two attachment housing materials (polymeric and metallic), cemented together with a dual-cure resin cement, were evaluated (Table 1). A total of 30 specimens were fabricated and tested. They were digitally designed using a CAD software program (SolidWorks 2023; Dassault Systèmes, Vélizy-Villacoublay, France) as square blocks measuring 14.5 × 14.5 × 4 mm. A centrally positioned Ø6.5 mm cylindrical cavity with a depth of 2.5 mm was designed to accommodate the attachment housings and to provide a uniform circumferential resin cement space. The cavity diameter was selected to replicate the clinical condition in which a Ø5.5 mm space was required for the attachment housing and an additional 0.5 mm circumferential space surrounding the housing was required for the securing material. Four auxiliary stabilization openings were also designed at the corners of each specimen to facilitate secure mounting and alignment on the testing platform. The standard tessellation language (STL) design files were exported to a 5-axis milling machine (DWX-52 DC; DGSHAPE Corp, Hamamatsu, Japan) and we used an appropriate CAM software (MillBox CAM 2020 edition; DGSHAPE Corp). The specimens for the 3 respective denture-base material groups were milled from prefabricated PMMA discs (breCAM.base; Bredent GmbH & Co. KG, Senden, Germany) and prefabricated PEEK discs (breCAM.BioHPP; Bredent GmbH & Co. KG), and they were additively manufactured using a cobalt-chromium (Co-Cr) alloy powder (AUDENTAL SLM POWDER; Audental Bio-material Co., Ltd., Shanghai, China) with a selective laser melting (SLM) system (EOSINT M 270; EOS GmbH, Krailling, Germany).

A stud-type implant-attachment system (Novaloc; Institut Straumann AG, Basel, Switzerland) with titanium (Ti) and PEEK housing materials was used. The housings were positioned at the center of the cavity using a positioning jig to verify the 0.5 mm space for the securing material. Prior to the cementation, the internal cavity surfaces of all specimens were airborne-particle abraded with 110 μm aluminum oxide (AL_2_O_3_) particles at 0.2 MPa for 10 s from 10 mm at a 45degree angle, and a composite resin primer (visio.link; bredent GmbH & Co. KG) was applied to the PMMA and PEEK specimens. A confocal microscope (μSURF; NanoFocus AG, Oberhausen, Germany) was used to assess the surface roughness profiles. A single-component adhesive priming material was applied (Clearfil Ceramic Primer Plus; Kuraray Noritake Dental Inc., Tokyo, Japan) to the internal cavity surfaces of the Co-Cr specimens. All tested components were bonded with a dual-polymerized resin cement (Panavia V5; Kuraray Noritake Dental Inc.). A vertical force of 2 N was applied during cementation to ensure consistent seating and cement thickness across all specimens, in accordance with standardized laboratory bonding protocols [21]. Excess cement was removed after it was initially polymerized using blue light in the range of 430 to 480 nm (Elipar Deep Cure I; 3M ESPE). Subsequently, the specimens were allowed to autopolymerize at 23 ± 2 °C and 65 ± 15% relative humidity for 6 min. The specimens were then stored in the same laboratory conditions for 24 h before mechanical testing.

A power analysis was performed using a software program (G Power version 3.1.9.7; Heinrich Heine University) to verify the sample-size adequacy. The selected sample size (n = 30) provided >95% power to detect large-to-very-large effects (α = 0.05), consistent with established laboratory protocols, while remaining feasible for standardized specimen fabrication and testing.

**Figure 1:**
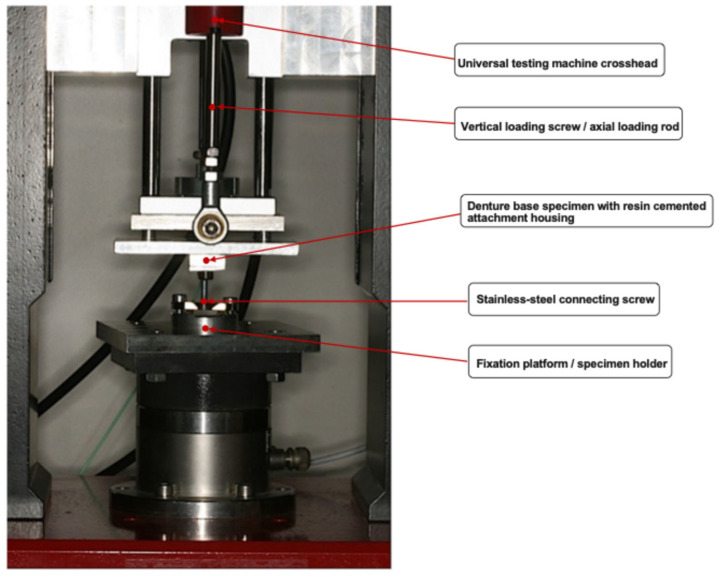
Experimental setup for the cyclic tensile-compressive loading.

Mechanical testing was performed using a universal testing machine (Micro NXG; Impact-BZ Ltd., London, UK) capable of performing cyclic loading movements (Figure 1). Prior to mechanical testing, the processing inserts from the attachment housings were removed, and the internal surfaces of the housings were rigidly connected to the tips of stainless-steel screws with a Ø5 mm shaft using a 2-component epoxy adhesive (EPOXY METAL; Bolton Adhesives, Rotterdam, The Netherlands). The 30 attachment-housing denture-base complexes were allocated to 6 groups (Groups A–D; n = 6 each, Groups E–F; n = 3 each) based on combinations of the tested materials (Table 2). Each specimen was subjected to 1100 cycles of force-controlled cyclic loading, consisting of alternating tensile and compressive phases. The number of cycles was selected to simulate repeated insertion–removal movements corresponding approximately to 1 year of clinical overdenture use, assuming 3 daily seating events [22]. Force data for both the tensile and compressive phases were recorded for every cycle. The loading frequency was controlled by testing software (Impact-bz; Impact-BZ Ltd.) and remained constant throughout the test. Failure was defined as the complete debonding of the attachment housing from the denture base, which automatically terminated the test. For each specimen, the maximum tensile force recorded during the 1100 cycles was used in the analysis. After completion of the mechanical testing, all specimens were examined under a stereomicroscope with ×10 magnification (BH2; Olympus Corp, Tokyo, Japan) to identify cohesive or adhesive failures based on the predominant location of the cement remnants. Scanning electron microscopy (SEM) was used to identify cement remnants on the surfaces of the housings after fracture using an SEM microscope equipped with energy dispersive spectroscopy (EDS) (JEOL JSM840A; Jeol Ltd., Akishima, Tokyo, Japan).

Because of the small sample size and unequal group distribution, the maximum force data were analyzed using nonparametric methods. Associations between the debonding occurrences and material variables were evaluated using Fisher’s exact test. Differences in the maximum tensile force reached during cyclic loading were assessed using the Kruskal–Wallis test for multiple group comparisons and the Mann–Whitney U test for pairwise comparisons. Exact *p*-values were reported where available. The effect sizes were calculated to aid interpretation of the magnitude of group differences. Statistical analysis was performed using a software program (IBM SPSS Statistics, v29.0; IBM Corp, Armonk, NY, USA), with significance set at α = 0.05.

## 3. Results

**Figure 2:**
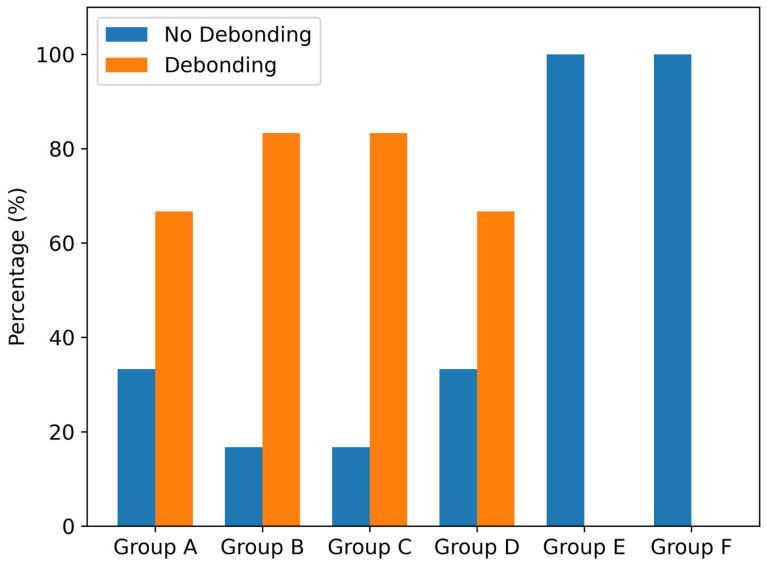
Debonding occurrence (%).

During cyclic mechanical loading, 18 out of 30 (60%) specimens debonded, whereas 12 out of 30 (40%) specimens completed all 1100 cycles without debonding (Figure 2). Debonding occurrence differed significantly among the denture-base materials (*p* = 0.004), but not significantly among the attachment-housing materials (*p* = 1.000). None of the specimens fabricated from the Co-Cr alloy debonded during cyclic mechanical loading. In contrast, debonding was observed in 75% of the specimens fabricated from the PMMA and in 75% from the PEEK denture-base materials (Table 3). When debonding was analyzed as a function of the combined effects of denture-base material, fabrication, surface-treatment combinations, and attachment-housing material, the results were statistically significant (*p* = 0.041). Debonding occurred exclusively in the PMMA and PEEK denture-base specimens, whereas no debonding was observed in the Co-Cr specimens, regardless of the housing material. Among the debonded specimens, most failures in the PMMA and PEEK groups occurred early during cyclic loading, with a median cycle-to-debonding of two cycles among the failed specimens. However, three PMMA specimens and three PEEK specimens completed the full 1100-cycle protocol without debonding. In addition, one PMMA and one PEEK specimen showed delayed debonding at 112 and 268 cycles, respectively. All Co-Cr specimens completed the 1100-cycle protocol without debonding. All failures were classified as adhesive because cement remnants were consistently detected on the attachment-housing surfaces and were absent from the denture-base cavities.

**Figure 3:**
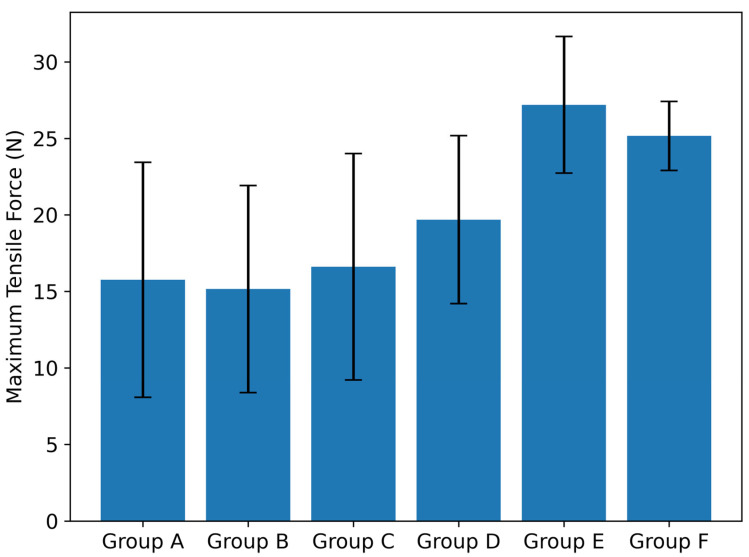
Boxplots of the maximum tensile loads (N).

The maximum tensile force measured during cyclic mechanical loading differed significantly among the three groups of denture-base materials, fabrication, and surface-treatment combinations (*H* [2] = 7.65, *p* = 0.022, η^2^ = 0.209). The specimens fabricated from the Co-Cr alloy demonstrated higher mean tensile-force values compared with the specimens fabricated from the PMMA and PEEK denture-base materials. A post hoc Bonferroni analysis revealed that the Co-Cr specimens exhibited significantly higher maximum tensile-force values than the PMMA (*p* = 0.005) and PEEK (*p* = 0.045) specimens. No statistically significant difference was observed between the PMMA and PEEK specimens (*p* = 0.890) (Table 4). No statistically significant main effect of attachment-housing material was detected for maximum force. Attachment-housing material was not significantly associated with debonding occurrence according to the Fisher’s exact test (*p* = 1.000). The Ti and PEEK attachment housings did not differ significantly according to the Mann–Whitney U test (U = 100.00, Z = −0.518, exact *p* = 0.624, r = 0.095). When the six-attachment-housing denture-base material combinations were compared separately, no statistically significant difference in maximum force was detected by the Kruskal–Wallis analysis (H(5) = 8.53, exact *p* = 0.119, η^2^ = 0.147) (Table 5, Figure 3).

**Figure 4:**
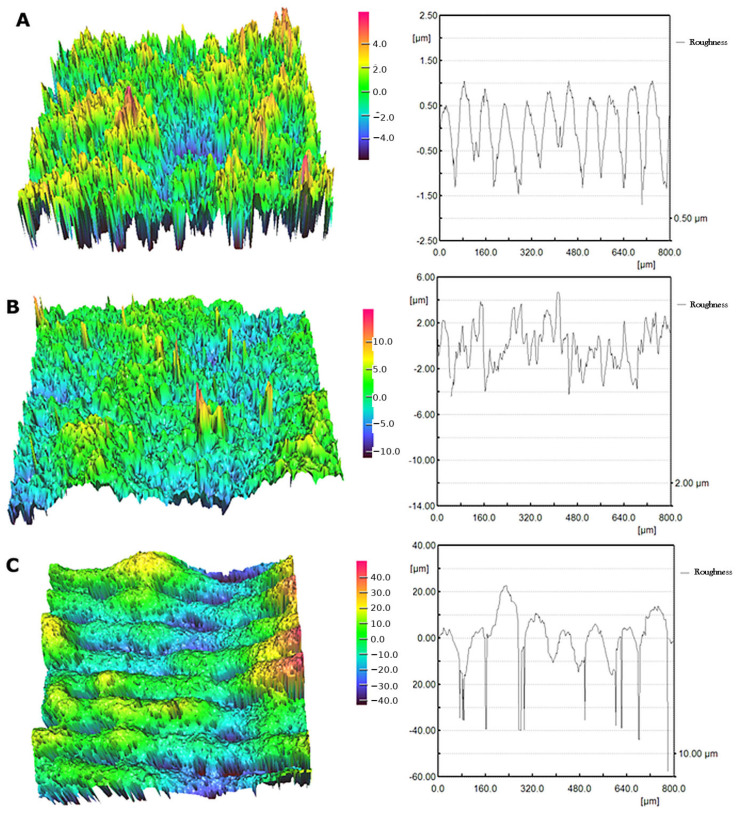
Representative confocal microscopy surface profiles of the denture-base materials after airborne-particle abrasion. (**A**) PEEK, (**B**) PMMA, and (**C**) Cr-Co.

The surface profiles of the bases revealed differences in specimen roughness. Specifically, the mean Sa value was 1.49 μm for the PEEK bases, 1.62 μm for the PMMA bases, and 8.22 μm for the Co–Cr bases. Indicative images are presented in Figure 4.

**Figure 5:**
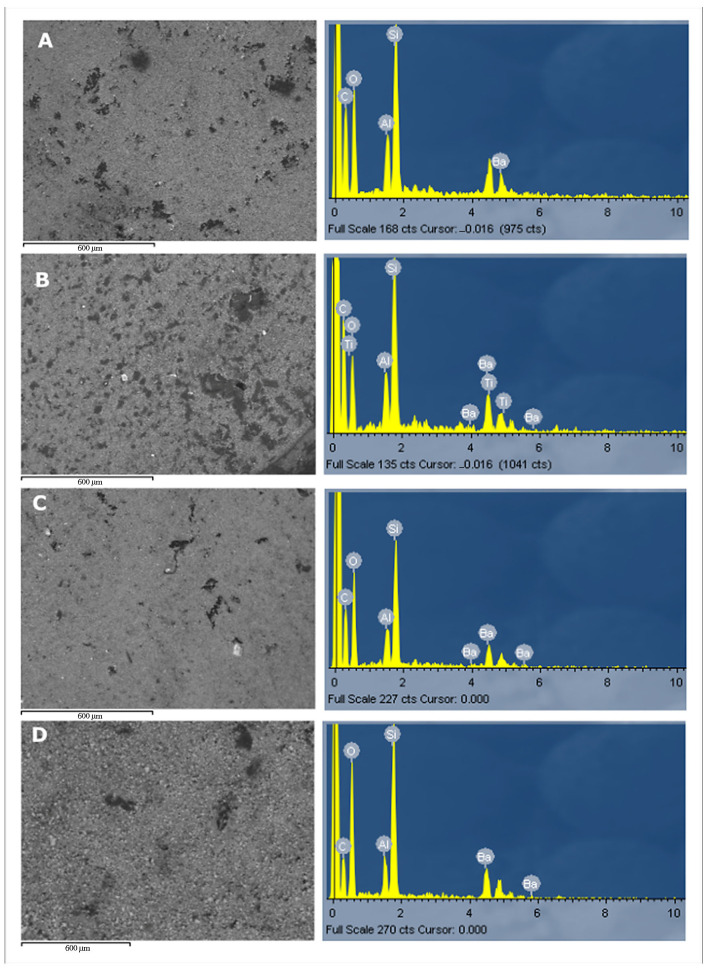
Representative post-fracture SEM–EDS evaluation and corresponding EDS spectra of the debonded specimens. (**A**) PEEK housing debonded from the PEEK base, (**B**) Ti-housing debonded from the PEEK base, (**C**) PEEK housing debonded from the PMMA base, and (**D**) Ti-housing debonded from the PMMA base.

The post-fracture SEM–EDS evaluation revealed cement remnants on the debonded housing surfaces of all the PEEK and PMMA specimens (Figure 5), while the corresponding base surfaces were free of detectable cement residues. This pattern suggested adhesive failure at the base–cement interface, with cement retention occurring mainly on the housing side. The detected Si, Ba, and Al were consistent with the inorganic filler phase of PANAVIA V5 resin cement. Underlying Ti was detected on the spectrum of the Ti housing for the specimens with the PEEK base (Figure 5B). In the Ti housing from the PEEK-base specimens, Ti was also detected in the SEM–EDS spectrum, indicating local exposure of the underlying titanium substrate (Figure 5B).

## 4. Discussion

In the present in vitro study, denture-base material, fabrication, and surface-treatment combinations significantly influenced both the occurrence of debonding and the maximum tensile force measured during cyclic loading. In contrast, attachment-housing material did not demonstrate a statistically significant effect on either debonding occurrence or maximum tensile force. A statistically significant interaction effect between denture-base material, fabrication, surface-treatment combinations and attachment-housing material was observed for debonding occurrence, but not for maximum tensile force. Accordingly, the null hypothesis was rejected with respect to denture-base material, fabrication, and surface-treatment combinations for both debonding occurrence and maximum tensile force, whereas it was not rejected with respect to attachment-housing material. Regarding the interaction between the attachment-housing and denture-base materials, the null hypothesis was rejected for debonding occurrence but not for maximum tensile force.

Most previously published investigations evaluating attachment-housing fixation have employed monotonic push-out or shear testing protocols [15,17,18,20] in which a direct force is applied to the housing until failure occurs, frequently requiring hollowing or sectioning of specimens to permit internal load transmission. Although such approaches quantify ultimate bond-strength under single-load conditions, they are destructive and may alter stress distribution within the attachment-housing–denture-base complex. In contrast, the present study employed cyclic axial tensile-compressive loading of intact housing–base complexes without subsequent monotonic pull-out testing of the non-debonded specimens. This experimental testing protocol may better simulate fatigue-related debonding behavior under repeated loading than ultimate adhesive bond-strength testing. Because the loading configurations and stress distributions differed substantially across the studies, the reported tensile force values should not be compared numerically with push-out data.

From a clinical standpoint, overdenture retention must balance stability and patient comfort. A retention range of approximately 4–20 N per attachment has been suggested as sufficient to provide functional security, while allowing manageable insertion and removal [11]. In addition, Novaloc retentive inserts are available in nominal retention values ranging from approximately 3 N to 25.5 N, depending on the insert type [13]. Within this mechanical framework, the bond strength of the attachment-housing–denture-base complex should ideally exceed the retentive force of the retentive insert. If the adhesive interface between the attachment housing and the denture base exhibits lower resistance than the insert retention, functional loading or overdenture removal may result in debonding of the housing rather than controlled retentive insert wear or replacement. The maximum tensile force values recorded in the present study fell within or above the clinically relevant retention range. However, the occurrence of debonding in both polymeric groups under cyclic loading suggested that fatigue degradation of the adhesive interface may have compromised long-term mechanical stability even when the initial housing retention appeared clinically acceptable.

Denture-base material, fabrication, and surface-treatment combinations significantly influenced the debonding behavior and force values. The PMMA-based specimens demonstrated lower mean maximum tensile values and higher failure incidence compared with the Co-Cr groups. The inferior performance of the PMMA specimens was likely attributable to the characteristics of the adhesive interface between the prepolymerized acrylic resin and the composite-based resin cement used. Although MMA-containing primers such as visio.link are intended to enhance bonding between polymeric and resin-based materials, adhesion between fully polymerized PMMA and highly cross-linked composite resin cement is primarily micromechanical rather than chemical [10]. Autopolymerizing acrylic pickup resin materials may exhibit greater chemical compatibility with PMMA due to shared methacrylate chemistry, whereas composite resin cements rely largely on surface roughening and primer-mediated interactions [17]. Limited potential for interdiffusion or covalent bonding at the PMMA–resin-cement interface may render this junction more susceptible to cyclic fatigue degradation.

Failure mode analysis further supported this interpretation. In all debonded specimens, cement remnants were observed adherent to the attachment-housing surfaces, while no cement remained within the denture-base cavity. The SEM–EDS analysis confirmed that these remnants contained Si, Ba, and Al, which are elements consistent with the inorganic filler phase of PANAVIA V5 (Kuraray Noritake Dental Inc., Tokyo, Japan) resin cement. This consistent pattern indicated that adhesive failure occurred predominantly at the denture-base–resin-cement interface rather than at the attachment-housing–resin-cement interface. The surface-roughness findings provided a complementary explanation, as the Co–Cr specimens exhibited substantially higher Sa values than the PMMA and PEEK specimens, which may have enhanced micromechanical interlocking with the resin cement and contributed to the absence of debonding in the Co–Cr groups. In contrast, the lower roughness of the PMMA and PEEK bases, combined with the limited potential for chemical bonding between the highly polymerized CAD–CAM polymers and resin cement, likely rendered the polymeric base–cement interface more susceptible to fatigue degradation under cyclic loading. No statistically significant main effect of attachment-housing material was detected. However, the effect size suggested a potentially meaningful difference among the combinations, which should be interpreted with caution due to the small and unequal group sizes. Because the same resin cement was applied across all groups, this absence of difference suggests that the housing material did not substantially influence the bond performance under the tested conditions. This finding should not be interpreted as evidence of equivalence between the Ti and PEEK housings, as equivalence or non-inferiority testing with predefined margins was not performed. Both the Ti and PEEK housings incorporate retentive, machined, macro-grooved surface designs, and the bonding mechanism under these conditions was likely governed primarily by micromechanical retention rather than by material-specific chemical adhesion. This finding contrasted with a similar study, in which the authors reported significant differences between Ti and PEEK housings in tensile force values. The increased tensile bond strength of the Ti housings could be attributed to differences in testing methodology, as no fatigue-related protocols were employed [19].

The cycle-to-debonding data provide additional insight into the failure pattern. Among the specimens that debonded, many PMMA and PEEK specimens failed during the early phase of cyclic loading, suggesting that failure in these specimens may have been related to initial interfacial instability rather than only to progressive fatigue accumulation. This interpretation is consistent with the failure mode analysis, in which cement remnants were observed on the attachment-housing surface and were absent from the denture-base cavity, indicating adhesive failure predominantly at the denture-base–resin-cement interface. However, the fact that three PMMA and three PEEK specimens completed the full protocol without debonding indicates that the polymeric denture-base materials were not inevitably associated with immediate failure. Rather, the findings suggest variability in the interfacial performance, possibly related to local differences in surface pretreatment effectiveness, primer interaction, cement adaptation, micromechanical retention, and initial interfacial integrity. The two delayed failures at 112 and 268 cycles further support variability in interfacial durability. All Co-Cr specimens completed the full protocol without debonding, but this finding should be interpreted within the limitations of the smaller Co-Cr sample size and the absence of residual pull-out testing after cyclic loading.

In the present study, standardized digital specimen fabrication ensured consistent geometry and cement thickness. Cyclic tensile-compressive loading provided a fatigue-oriented assessment of attachment-housing–denture-base behavior, and the evaluation of both debonding occurrence and maximum tensile force offered complementary mechanical endpoints. Certain limitations must also be acknowledged. This was an in vitro study conducted under controlled laboratory conditions, where intraoral factors such as thermal cycling, moisture exposure, pH variation, enzymatic degradation, microbial colonization, chemical challenges from food and beverages, and multidirectional loading were not simulated. The loading protocol was purely axial and did not incorporate lateral or rotational, bending, or impact forces. Overdentures are exposed not only to insertion-removal events but also to masticatory, lateral, rotational, and multidirectional functional loading, and so the results should be interpreted as reflecting the tested tensile-compressive cyclic condition should not be interpreted as a complete simulation of one year of total oral function. The non-debonded specimens were not subjected to subsequent monotonic pull-out testing, and residual bond strength following cyclic loading was not determined. Additionally, only one resin cement system and surface-treatment protocol were evaluated. Alternative adhesive systems specifically designed to enhance polymer–polymer chemical interaction may demonstrate different mechanical behaviors. Furthermore, the unbalanced group distribution, particularly the smaller number of Co-Cr specimens, may have reduced the statistical power for detecting interaction effects between the denture-base material and attachment-housing material. Therefore, interaction-related findings should be interpreted cautiously.

Future investigations should evaluate the same material combinations in larger and more balanced datasets after incorporating thermomechanical aging, artificial saliva storage, pH cycling, microbial biofilm exposure, and multidirectional loading to better simulate intraoral conditions and determine whether environmental degradation alters debonding behavior. A comparative evaluation of composite resin cements, autopolymerizing acrylic pickup resins, and adhesive systems formulated for improved bonding to polymeric denture bases warrants further exploration, with a potential complementary finite element analysis. Ultimately, prospective clinical studies are needed to determine whether the fatigue resistance observed in the metallic denture base frameworks could translate into improved long-term clinical performance of implant-supported overdentures.

## 5. Conclusions

Within the limitations of this in vitro study, the following conclusions were drawn:

1. Denture-base material, fabrication, and surface-treatment combinations significantly influenced the debonding occurrence of resin-cemented attachment-housing–denture-base complexes under cyclic mechanical loading.
2. The Co-Cr denture-base specimens did not exhibit debonding within the applied cyclic loading protocol, possibly related to their higher surface roughness and enhanced micromechanical retention of the resin cement, whereas debonding was frequently observed in the PMMA and PEEK denture-base specimens.
3. Denture-base material, fabrication, and surface-treatment combinations significantly affected the maximum tensile force measured during cyclic mechanical loading, with the Co-Cr specimens demonstrating higher values than the PMMA and PEEK specimens.
4. No statistically significant main effect of attachment-housing material was detected, but this should not be interpreted as evidence of equivalence between the titanium and PEEK housings.

## Figures and Tables

**Table 1:** Study materials used.

Material	Type	Manufacturer	Ref Number
breCAM.base	Polymethylmethacrylate (PMMA) milling disc	Bredent GmbH & Co. KG	54PC2030
breCAM.BioHPP	Polyetheretherketone (PEEK) milling disc	Bredent GmbH & Co. KG	54002029
Audental SLM Powder	Cobalt-chromium (Co-Cr) alloy powder	Audental Bio-material Co., Ltd.	AU-CCP-000
Novaloc Matrix System	Titanium (Ti) housing	Institut Straumann AG	2010.701-NOV
Novaloc Matrix System	Polyetheretherketone (PEEK) housing	Institut Straumann AG	2010.702-NOV
PANAVIA V5 Paste	Dual-polymerized resin cement	Kuraray Noritake Dental Inc.	#3611-EU
Clearfil Ceramic Primer Plus	Single-component adhesive primer	Kuraray Noritake Dental Inc.	#3637-EU
visio.link	Composite resin primer	Bredent GmbH & Co. KG	VLPMMA10

**Table 2:** Evaluated specimen groups.

	Specimens (n)	Denture-Base Material	Attachment-Housing Material
Group A	6	PMMA ^1^	Ti ^4^
Group B	6		PEEK
Group C	6	PEEK ^2^	Ti
Group D	6		PEEK
Group E	3	Co-Cr ^3^	Ti
Group F	3		PEEK

^1^ PMMA, polymethylmethacrylate; ^2^ PEEK, polyetheretherketone; ^3^ Co-Cr, cobalt-chromium; ^4^ Ti, titanium.

**Table 3:** Frequency distribution of debonding occurrence.

(n *)	Group A (6)	Group B (6)	Group C (6)	Group D (6)	Group E (3)	Group F (3)	Total (30)
No debonding	33.3% (2)	16.7% (1)	16.7% (1)	33.3% (2)	100% (3)	100% (3)	40% (12)
Debonding	66.7% (4)	83.3% (5)	83.3% (5)	66.7% (4)	0% (0)	0% (0)	60% (18)

* n; specimen number.

**Table 4:** Bonferroni post hoc test for the denture-base materials.

(I) Material	(J) Material	Mean Difference (I − J)	Standard Error	*p* *-Value	95% Confidence Interval	
					Lower Bound	Upper Bound
PMMA ^1^	PEEK	−2.689	2.527	0.89	−9.139	3.761
	Co-Cr	−10.725	3.095	**0.005**	−18.625	−2.825
PEEK ^2^	PMMA	2.689	2.527	0.89	−3.761	9.139
	Co-Cr	−8.036	3.095	**0.045**	−15.936	−0.136
Co-Cr ^3^	PEEK	8.036	3.095	**0.045**	0.136	15.936
	PMMA	10.725	3.095	**0.005**	2.825	18.625

^1^ PMMA, polymethylmethacrylate; ^2^ PEEK, polyetheretherketone; ^3^ Co-Cr, cobalt-chromium; * *p* < 0.05; statistically significant *p*-values are shown in bold.

**Table 5:** Descriptive statistics for the maximum tensile-force measurements.

Groups	N	Mean	±SD	Minimum	Maximum	Median	IQR
A	6	15.76	7.68	6.38	25.81	14.9	15.37
B	6	15.16	6.77	8.59	26.54	14.29	10.49
C	6	16.61	7.4	8.54	26.52	17.01	13.7
D	6	19.69	5.49	13.77	26.79	17.39	10.65
E	3	27.2	4.47	23.6	32.2	25.8	NR
F	3	25.17	2.26	23	27.5	25	NR
Total	30	18.68	7.19	6.38	32.2	17.72	13.2

NR, Not Reported.

## Data Availability

The data supporting the findings of this study are available from the corresponding author upon reasonable request.
